# Smart EV charging via advanced ongrid MPPT-PV systems with quadratic-boost split-source inverters

**DOI:** 10.1038/s41598-025-90775-w

**Published:** 2025-03-06

**Authors:** Mostafa Wageh Lotfy, Haitham S. Ramadan, Sherif M. Dabour

**Affiliations:** 1 Dept. of Process Control Technology, Faculty of Technology and Education, Beni-Suef University, Beni-Suef, Egypt; 2 Electrical Power and Machines Dept, Faculty of Engineering, Zagazig University, Zagazig, 44519 Egypt; 3 ISTHY, Institut National du Stockage Hydrogène, Morvillars, Belfort Territory, 90120 France; 4 Department of Electrical Power and Machines Engineering, Tanta University, Tanta, 31733 Egypt

**Keywords:** Grid connection, Renewable Energy, Three-phase split-source inverter (SSI), Quadratic-Boost SSI, Electric Vehicle, Renewable energy, Electrical and electronic engineering, Energy infrastructure

## Abstract

This paper presents an enhanced Maximum Power Point Tracking (MPPT) algorithm for Quadratic-Boost Split Source Inverters (QB-SSI), designed for grid-connected Photovoltaic (PV)-powered smart charging stations for Electric Vehicles (EVs). The proposed algorithm integrates advanced control strategies and adaptive techniques to address the limitations of traditional MPPT techniques. By dynamically adjusting to operating conditions and environmental factors, the enhanced algorithm achieves accurate and rapid tracking of the maximum power point (MPP) under dynamic conditions. The paper provides a comprehensive overview of the QB-SSI topology, a detailed mathematical model, and simulation results. These findings demonstrate the superior performance of the enhanced MPPT algorithm, offering significant improvements in tracking accuracy, convergence time, and efficiency, thereby enhancing the overall performance of QB-SSI-based PV systems. A modified space vector modulation (SVPWM) is utilized to enhance the inverter switching characteristics and dc-boosting capability. The overall system is modeled via MATLAB/Simulink^™^, and the experimental results providing valuable insights into the performance and functionality of the proposed algorithm. This allows for a comprehensive analysis of its capabilities and potential advantages in practical applications.

## Introduction

PHOTOVOLTAIC (PV) systems have emerged as a promising renewable energy source due to their potential for providing clean and sustainable electricity^1^. The rapid decline in the cost of PV modules, along with technological advancements in power electronics, has resulted in the widespread implementation of PV systems worldwide. Despite these advances, the efficiency of PV systems is still a major concern, as the output power of a PV module is highly dependent on various factors such as solar irradiance, temperature^2,3^, and the angle of incidence. Consequently, researchers have proposed various Maximum Power Point Tracking (MPPT) algorithms to improve the efficiency and performance of PV systems, thereby increasing the output power extracted from the solar panels^4,5^.

**Fig. 1:**
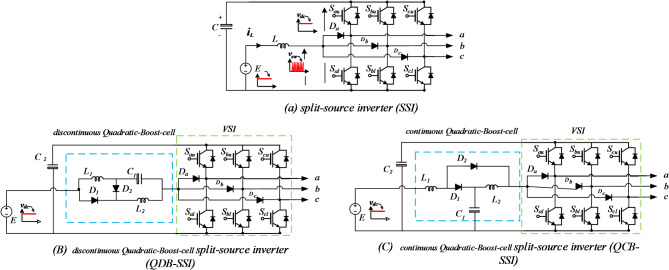
SSI inverter and Quadratic-Boost SSI.

The split-source inverter (SSI) topology stands out, particularly in its three-phase configuration^6^, achieved by integrating a DC-boost converter into the conventional three-phase voltage source inverter (VSI). This integration involves connecting the boost inductor to the AC output terminals of inverter legs a, b, and c through diodes denoted as $\:{D}_{a,}{D}_{b,}\:and\:{D}_{c}$, as illustrated in Fig. 1a^7,8^. Among the realm of photovoltaic (PV) systems, the inverter serves as a critical component that perform the boosting of DC Voltage and converting it into alternating current (AC) power for grid feeding or local consumption. Among the recent inverter topologies, the Quadratic-Boost Split Source Inverter (QB-SSI), shown in Fig. 1b and c has garnered attention for its advantages in voltage gain, reduced voltage stress on switches, and enhanced efficiency^9,10^. By amalgamating the characteristics of quadratic boosting and dc-ac conversion, the QB-SSI achieves higher voltage gain with lower component stress compared to conventional topologies. However, the performance of the QB-SSI hinges on the efficacy of the Maximum Power Point Tracking (MPPT) algorithm employed, as it directly influences power transfer from PV panels to the inverter^11,12^.

The performance of PV systems hinges on optimal power extraction and conversion. MPPT strategies enhance energy output in variable conditions, while inverter configurations influence both efficiency and the stress on components. Table 1 details the advantages and drawbacks of prominent MPPT methods, including Perturb and Observe (P&O), Incremental Conductance (IncCond), Hybrid Techniques, and Machine Learning-Based MPPT, citing references^4,5,13–18^. Table 2 examines different inverter designs such as Two-Stage DC-DC-AC, Single-Stage DC-AC (VSI), and Single-Stage QB-SSI, evaluating aspects like voltage gain, component stress, and overall efficiency with references^6,7,9,10,19,20^.

**Table 1:** Comparison of MPPT techniques.

MPPT Technique	Strengths	Limitations	Addressed in Proposed Work	Convergence Speed	Steady-State Oscillations	References
Perturb and Observe (P&O)	Simple implementation, low cost	Oscillations around MPP, slower convergence	Improved convergence speed	Medium	High (oscillations near MPP)	^4,5^
Incremental Conductance (IncCond)	Accurate tracking, good for dynamic conditions	Complex implementation, computationally intensive	Simplified computation	Medium-Fast	Low	^13,14^
Hybrid Approaches	Combines strengths of multiple techniques	Higher complexity, costly	Adaptive strategy reduces complexity	Fast	Very Low	^15,16^
Machine Learning-Based MPPT	High accuracy under complex conditions	Requires training data, computationally intensive	Integrates adaptive learning for scalability	Fast	Minimal	^17,18^

This paper aims to refine the MPPT Algorithm for QB-SSI connected to grid systems in PV applications to boost both efficiency and performance, as indicated by reference^21^. The suggested algorithm leverages sophisticated control strategies and optimization techniques to accurately identify and follow the MPP of PV modules, ensuring efficient energy transfer to the QB-SSI, supported by reference^22^. Literature suggests various MPPT algorithms to improve PV system efficiency, divided into model-based and model-free categories.

Model-based methods, such as the Newton-Raphson technique and Maximum Power Line method, utilize mathematical modelling of PV modules to determine the MPP, referenced in^23–29^. On the other hand, model-free methods, including P&O and IncCond, iteratively adjust the operating parameters of PV modules based on real-time power output changes, referenced in^13,14,30,31^. Although simpler, model-free methods can experience oscillations around the MPP, which may lead to reduced efficiency and energy losses. To overcome these challenges, hybrid and adaptive approaches that merge model-based and model-free strategies have been developed to enhance accuracy and response times, utilizing advanced controls like fuzzy logic, neural networks, and genetic algorithms, as noted in^15–18,32,33^.

The QBSSI topology has been proposed as a potential solution to address the voltage gain and component stress limitations of traditional inverter topologies^19^. By integrating the characteristics of both the quadratic boost and split-source converters, the QBSSI offers a higher voltage gain and reduced voltage stress on the switches, which leads to improved efficiency and reliability^34,35^. However, the performance of the QBSSI is also influenced by the MPPT algorithm employed, as it determines the amount of power extracted from the PV panels and transferred to the inverter.

Battery Electric Vehicles (BEVs) depend on the electrical grid to fulfill their energy needs. Due to the high maximum charging power of BEVs compared to other household energy consumers, regulatory bodies and industry stakeholders have developed specific plug types (as defined in IEC 62196) and communication protocols to ensure the safe handling of high voltage and currents associated with BEV charging. Additionally, the Tesla Model 3 RWD, with a battery capacity of 50.0 kWh and an efficiency of 15.6 kWh/100 km, exemplifies this trend. Conductive charging systems, characterized by a cable-plug connection for electricity transmission, are widely adopted. In contrast, inductive charging, known as wireless charging, utilizes electromagnetic induction to transfer energy between induction coils^36–38^.

In this paper, we address a significant gap in the current research by focusing on the QB-SSI design, which uniquely integrates DC-boosting and inverter stages. This integration not only minimizes component stress but also enhances the suitability of QB-SSI for scalable applications. Furthermore, we contribute to the body of knowledge by presenting an adaptive Maximum Power Point Tracking (MPPT) algorithm specifically optimized for QB-SSI. This optimization is critical for extending the scalability of QB-SSI to accommodate large installations and high-capacity electric vehicle (EV) networks, demonstrating the potential for broad applicability in renewable energy systems.

## Topologies of quadratic-boost SSI

**Fig. 2:**
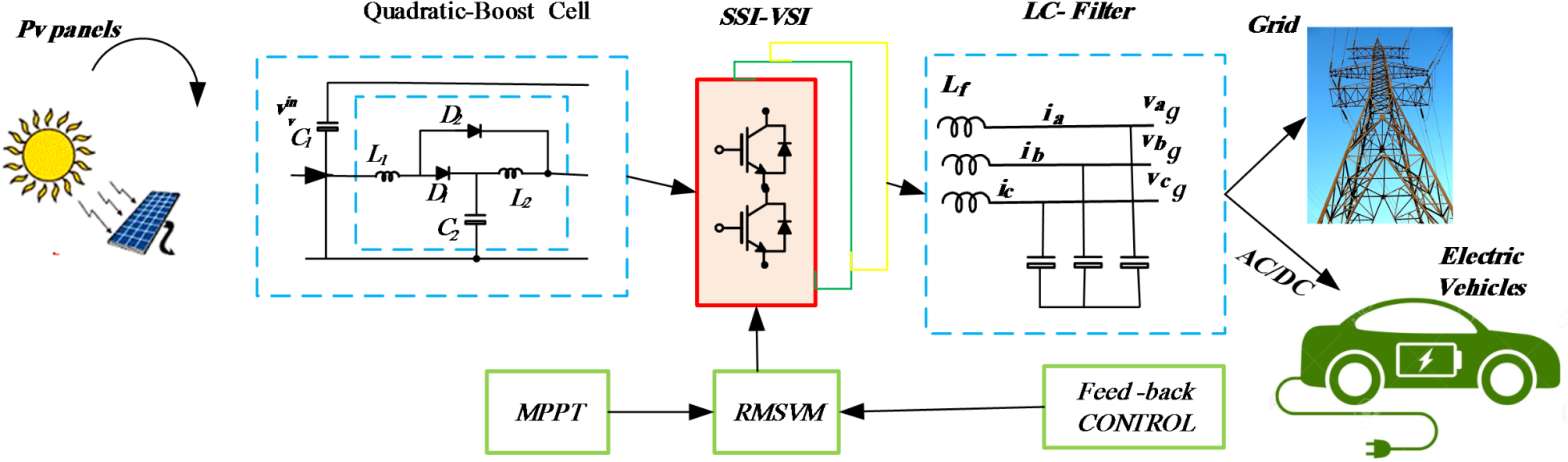
The circuit diagram of the QB-SSI network-dependent grid-connected PV system.

Figure 1 illustrates three distinct configurations of the proposed Quadratic Boost Inverter (QBI). In each configuration, the input inductance of the basic Single-Stage Inverter (SSI), denoted as $\:{L}_{1}$ in Fig. 1(a), is substituted with an impedance cell. This cell comprises two inductors ($\:{L}_{1}$ and $\:{L}_{2}$), a capacitor ($\:{C}_{1}$), and two diodes ($\:{D}_{1}$ and $\:{D}_{2}$). This arrangement enables cascaded boosting without the need for additional switching states. Figure 2 depicts the schematic of the proposed setup, consisting of five key components: PV panels, Quadratic-Boost Cell, filter, utility grid, and control unit. The control system includes three units: MPPT, outer control loop, and inner control loop.

The QBI depicted in Fig. 1(b) exhibits a discontinuous input current, unlike the other configurations shown in Fig. 1(c). The maintenance of a continuous input current is attained by integrating the input inductance $\:{L}_{1}$ in series with the power supply. By doing so, the supply current is buffered, and the associated stresses are reduced. On the flip side, the Quadratic Boost Inverter with discontinuous input current (DC-QBI), depicted in Fig. 1(b), requires an additional parallel capacitance connected to the power supply to alleviate the negative impact of input ripples. However, the voltage stress on the capacitor $\:{C}_{1}$ in the DC-QBI is less than that experienced in the Quadratic Boost Inverter with continuous input current (CC-QBI).

Furthermore, all the topologies share a common direct current (DC) rail connecting the power supply and the inverter bridge. This connection serves to minimize the impact of common-mode noise.

### Charging mode

The charging mode can be activated by turning ON at least one of the lower switches in the inverter bridge. When this happens, the voltage at the dc-side output becomes zero due to the presence of a short circuit. Moreover, diode $\:{D}_{1}$ becomes non-conductive, while diode $\:{D}_{2}$ starts conducting. Consequently, both inductors $\:{L}_{1}$ and $\:{L}_{2}$ receive a charge. Utilizing Kirchhoff’s Voltage Law (KVL), we can discern the subsequent steady-state relationships in the charging duty cycle, $\:{D}_{ch}$.

1 $$\:\left\{\begin{array}{l}{v}_{L1}=E\:\:\:\:\:\:\:\&\:\:\:\:{v}_{L2}={V}_{C1}\\\:{i}_{C1}=-{i}_{L2}\:\:\:\&\:\:\:{v}_{D1}={-V}_{L2}\\\:{i}_{ch}={i}_{L1}+{i}_{L2}\end{array}\right.$$

and

2 $$\:\left\{\begin{array}{l}{v}_{L1}=E\:\:\:\:\:\:\:\&\:\:\:\:{v}_{L2}=E+{V}_{C1}\\\:{i}_{C1}=-{i}_{L2}\:\:\:\&\:\:\:{v}_{D1}={-V}_{L2}\\\:{i}_{ch}={i}_{L1}+{i}_{L2}\end{array}\right.$$

### Discharging mode

The discharge mode is exclusively initiated when all the upper switches in $\:{B}_{6}\:$are turned ON. In this mode, diode $\:{D}_{1}\:$conducts while $\:{D}_{2}$ remains in a blocking state. This arrangement establishes the relationship during the discharging duty cycle, denoted as$\:{\:D}_{dis}\:=\:1\:-\:{D}_{ch}$.

3 $$\:\left\{\begin{array}{l}{v}_{L1}=E-{V}_{C1}\:\:\:\:\:\:\\\:{v}_{L2}={V}_{C1}-{V}_{C2}\\\:{i}_{C1}={i}_{L1}-{i}_{L2}\:\:\:\\\:{v}_{D2}={V}_{L2}\end{array}\right.$$

4 $$\:\left\{\begin{array}{l}{v}_{L1}=-{V}_{C1}\:\:\:\:\:\:\\\:{v}_{L2}=E+{V}_{C1}-{V}_{C2}\\\:{i}_{C1}={i}_{L1}-{i}_{L2}\:\:\:\\\:{v}_{D2}={V}_{L2}\end{array}\right.$$

The voltage across the capacitor $\:{C}_{1}$ in both configurations is expressed as:

5 $$\:\left\{\begin{array}{l}{v}_{C1}=\frac{E}{\left[1-{D}_{Ch}\right]}={{\upbeta\:}}_{\text{v}}E\:\:\to\:\:\left(DC-QBI\right)\:\:\:\:\\\:{v}_{C1}=\frac{E*{D}_{Ch}}{\left[1-{D}_{Ch}\right]}=\left({{\upbeta\:}}_{\text{v}}-1\right)E\:\to\:\left(DC-QBI\right)\end{array}\right.$$

From (5), it is clear that the voltage across capacitor $\:{C}_{1}$​ in the DC-QBI is significantly reduced in comparison to the CC-QBI configuration. Nonetheless, the voltage stress on the inverter switches, measured across capacitor $\:{C}_{2}$​, remains consistent for both configurations. The output ac voltage gain (G) is determined by (B)

6 $$\:{V}_{C2}=\frac{E}{{\left[1-{D}_{Ch}\right]}^{2}}={{\upbeta\:}}_{\text{v}}^{2}E\:$$

Here, $\:{\beta\:}_{v}$ represents the dc-boosting factor of the fundamental SSI, and its value is determined by the expression:

7 $$\:\begin{array}{c}{{\upbeta\:}}_{\text{v}}=\frac{1}{\left[1-{D}_{Ch}\right]}\:\:\:\end{array}$$

The output ac voltage gain (*G*) is determined by:

8 $$\:\begin{array}{c}\text{G}=\frac{{\widehat{V}}_{\varnothing\:1}}{E}=\:\frac{{M}_{ac}}{\sqrt{3}{[1-{D}_{Ch}]}^{2}}\:\:\end{array}$$

The fundamental peak output phase voltage, denoted as ^1, is of particular significance. It is worth emphasizing that for conventional (VSIs), the value of is constrained to be less than or equal to $\:1/\sqrt{3}$.

## Control of of quadratic-Boost SSI

In this section, a novel approach is introduced for enhancing the boosting capability of a single-stage DC-AC inverter. The proposed inverter topology extends the conventional split-source inverter by incorporating a cell, which consists of one capacitor, two inductors, and two diodes. By making use of the common-mode component present in the AC modulation signals, the decoupled control technique is employed to regulate the DC input current in this section of the paper, specifically for (QB-SSI).

**Fig. 3:**
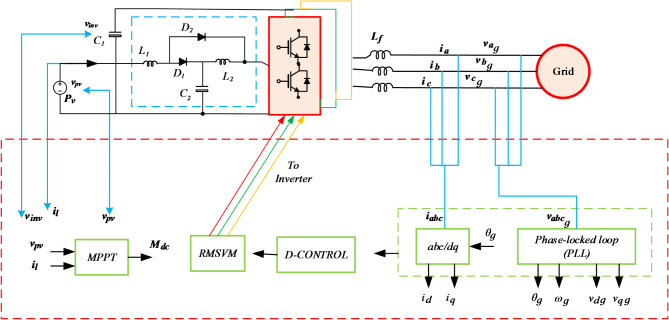
Closed-loop control configuration of the Proposed SSI connected to the grid.

The analysis focuses on the investigation and development of a control scheme for Single-Stage Inverter (SSI) in grid-connected mode. The main aim is to evaluate the suitability of employing the synchronous reference frame control technique in a two-stage configuration (conventional three-phase inverter). In this setup, the dc-link voltage is governed by the output current controller, while the input current or voltage is regulated by the duty cycle (*D*).To achieve this, the proposed topology for the SSI modulation scheme is presented, Introducing two control parameters that facilitate the independent regulation of both the AC and DC sides of the SSI within defined constraints, akin to other single-stage topologies. The decoupled closed-loop control strategy for the grid-connected QB-SSI is formulated by integrating the regulated modulation approach (RMSV) with the widely employed synchronous reference frame control technique. This control strategy employs the two control parameters derived from the RMSV modulation technique, encompassing an input power control component and a grid-side control component. The modulation index $\:{M}_{ac}\:$oversees the grid side, whereas the regulation index $\:{M}_{dc}\:$controls the DC side. The control method for the grid-connected Single-Stage Inverter (QBSSI) is presented in Fig. 3 and discussed in the subsequent sections within the rotating dq reference frame. The SSI is connected to the grid through an interface inductance Lf filter.

**Fig. 4:**
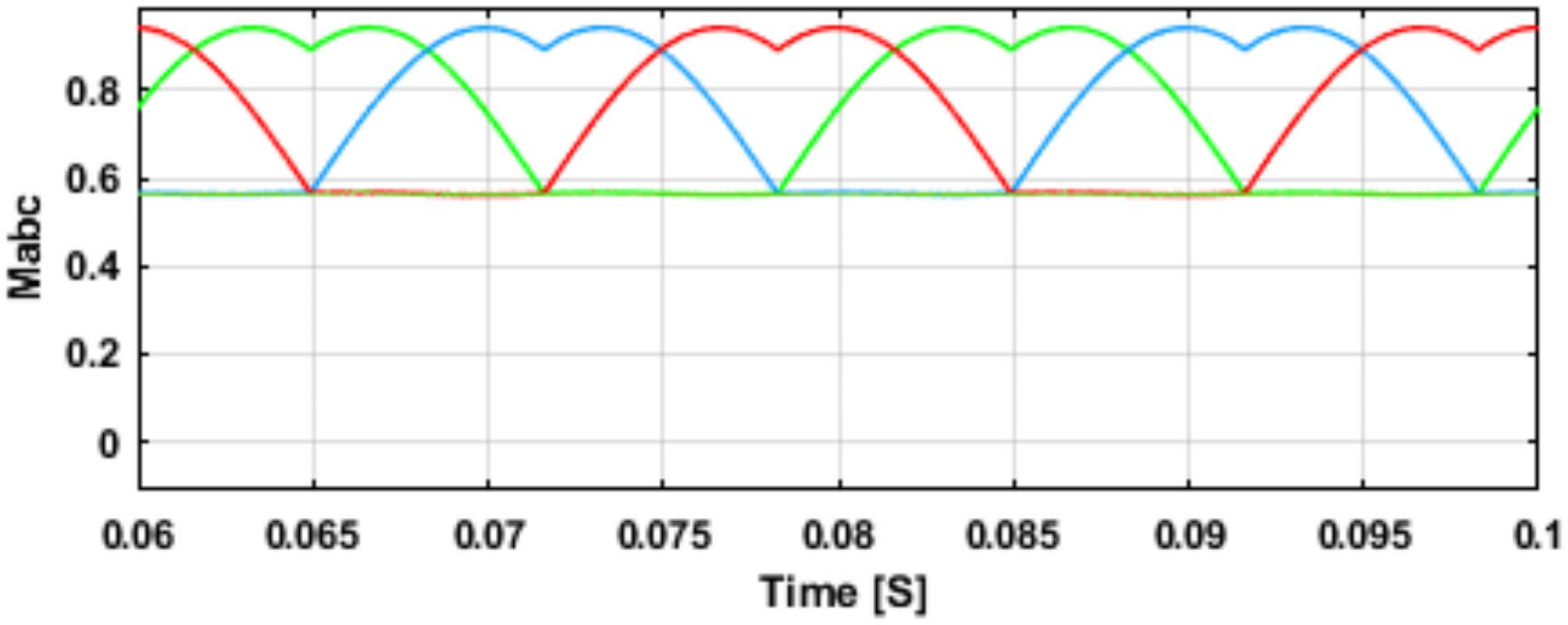
Modulating signals.

Figure 4 depicts the executed (RMSV) modulation, which entails the transformation of sinusoidal reference signals from the synchronous reference frame controller into space vector equivalent modulating signals and subsequent application of saturation. The resultant saturated space vector modulating signals are then converted into RMSV modulating signals, where the negative envelope (min ($\:{v}_{a}^{*}{,v}_{b}^{*}{,v}_{c}^{*}$*)) is set to zero, represented by $\:{M}_{ac}=1$. The input current controller modifies $\:{M}_{ac}$ in response to the input current reference, thereby adjusting the negative envelope.

The connection between the average DC-link voltage ($\:{v}_{inv}$) and the input DC voltage ($\:{v}_{inv}$), along with the output fundamental peak phase voltage ($\:{v}_{\phi\:1}$) as a function of ($\:{v}_{inv}$), is detailed in^16^.

9 $$\:{v}_{inv}=\frac{1}{1-{M}_{dc}}*{v}_{inv}$$

10 $$\:{v}_{\phi\:1}=\frac{{M}_{ac}}{\sqrt{3}}*{v}_{inv}$$

### MPPT controller

To maintain the PV operating point at the desired level, the quadratic-boost SSI utilizes the duty cycle $\:{M}_{dc}$. This duty cycle ensures that the PV voltage corresponds to $\:{V}_{mp}$, which represents the maximum available power considering the given temperature and radiation conditions. The value of $\:{V}_{mp}\:$can be determined using any Maximum Power Point Tracking (MPPT) technique^15^.

To achieve precise control over the PV voltage, cascaded controllers are employed, as shown in Fig. 3. The PV voltage $\:{V}_{pv}$, undergoes comparison with the reference value $\:{V}_{mp}$, and the resultant error is directed to a Proportional-Integral (PI) controller. The output of this controller signifies the current reference. Subsequently, this reference current is juxtaposed with the actual input current of the voltage-lift cell SSI, and the resulting error is supplied to another PI controller. This second PI controller generates the required duty cycle, $\:{M}_{dc}$, which is utilized by the RMSVM modulation technique.

### P&O-based MPPT technique

**Fig. 5:**
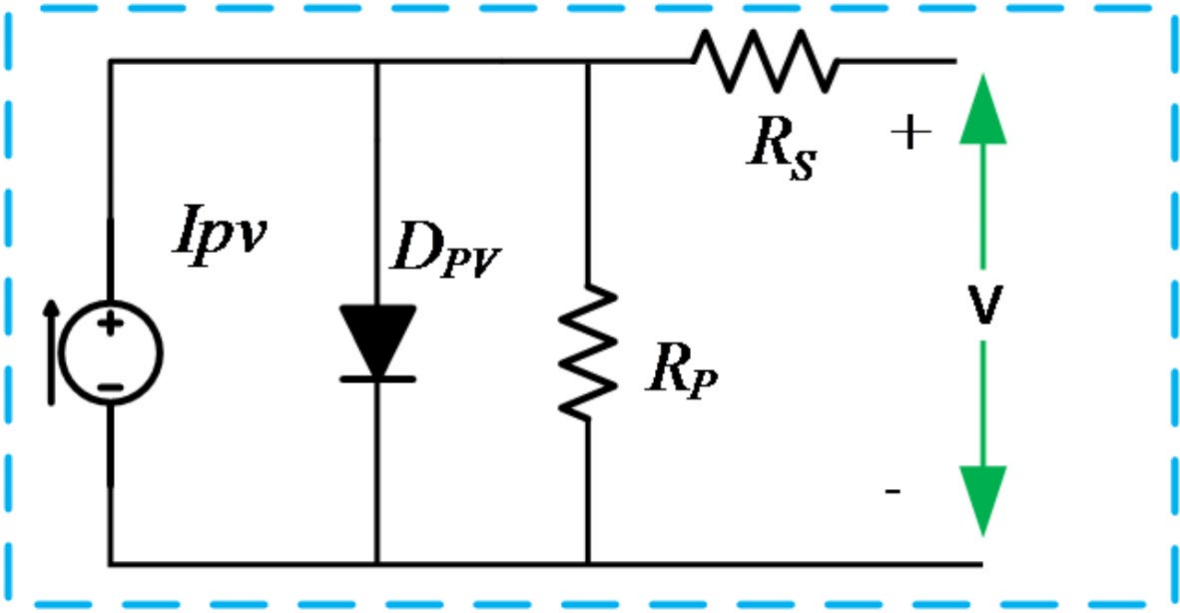
Equivalent circuit of a solar cell.

The PV cell’s mathematical model is depicted as a voltage-controlled current source, which is notably responsive to its input parameters, such as solar irradiation power (W/m2) and temperature (C°). An equivalent circuit representation of the photoelectric cell is commonly employed, as shown in Fig. 5^29^.

11 $$\:{I}_{pv}={I}_{ph}-{I}_{r}\left[\text{exp}\left(q\left(V+{R}_{s}{I}_{pv}\right)/\eta\:kT\right)-1\right]-\left(V+{R}_{s}{I}_{pv}\right)/{R}_{p}$$

where.

$\:{R}_{s},{R}_{p}$​: Series and parallel resistances.

$\:q$: Charge of an electron.

$\:V$: Voltage across the PV cell.

$\:T$: Temperature in Kelvin.

$\:\eta\:$: Diode ideality factor.

$$\:k$$: Boltzmann constant.

$\:{I}_{ph}$​: Photocurrent, which is determined from

12 $$\:I_{{ph}} = \left\lceil {I_{{sc}} + \alpha \:\left( {T - T_{{ref}} } \right)} \right\rceil \:\frac{N}{{1000}}$$

$\:{I}_{r}$​: Reverse saturation current and it is determined from

13 $$\:I_{r} \: = I_{{rr}} \left( {\frac{T}{{T_{{ref}} }}} \right)^{3} e^{{\left\lceil {\left( {qE_{g} /\eta \:k} \right)\left( {\left( {1/T_{{ref}} } \right) - \left( {1/T} \right)} \right)} \right\rceil }}$$

Here.

$$\:a$$: Constant.

$\:{E}_{g}$: Bandgap energy of the semiconductor material.

$\:{I}_{rr}$​: Reverse saturation current at $\:{T}_{ref}$​. It is determined from

14 $$\:{I}_{rr}\:=\frac{{I}_{sc}\:-({V}_{oc}/{R}_{p})}{\left[\text{exp}\left[\frac{q{V}_{oc}}{\eta\:k{T}_{ref}}\right]\right]-1}$$

The open-circuit voltage depends on the temperature $\:T$ and varies with the coefficient $\:\beta\:$ as follows:

15 $$\:{V}_{oc}\left(T\right)={V}_{oc}\left({T}_{ref}\right)+\beta\:\left(T-{T}_{ref}\right)\:$$

**Fig. 6:**
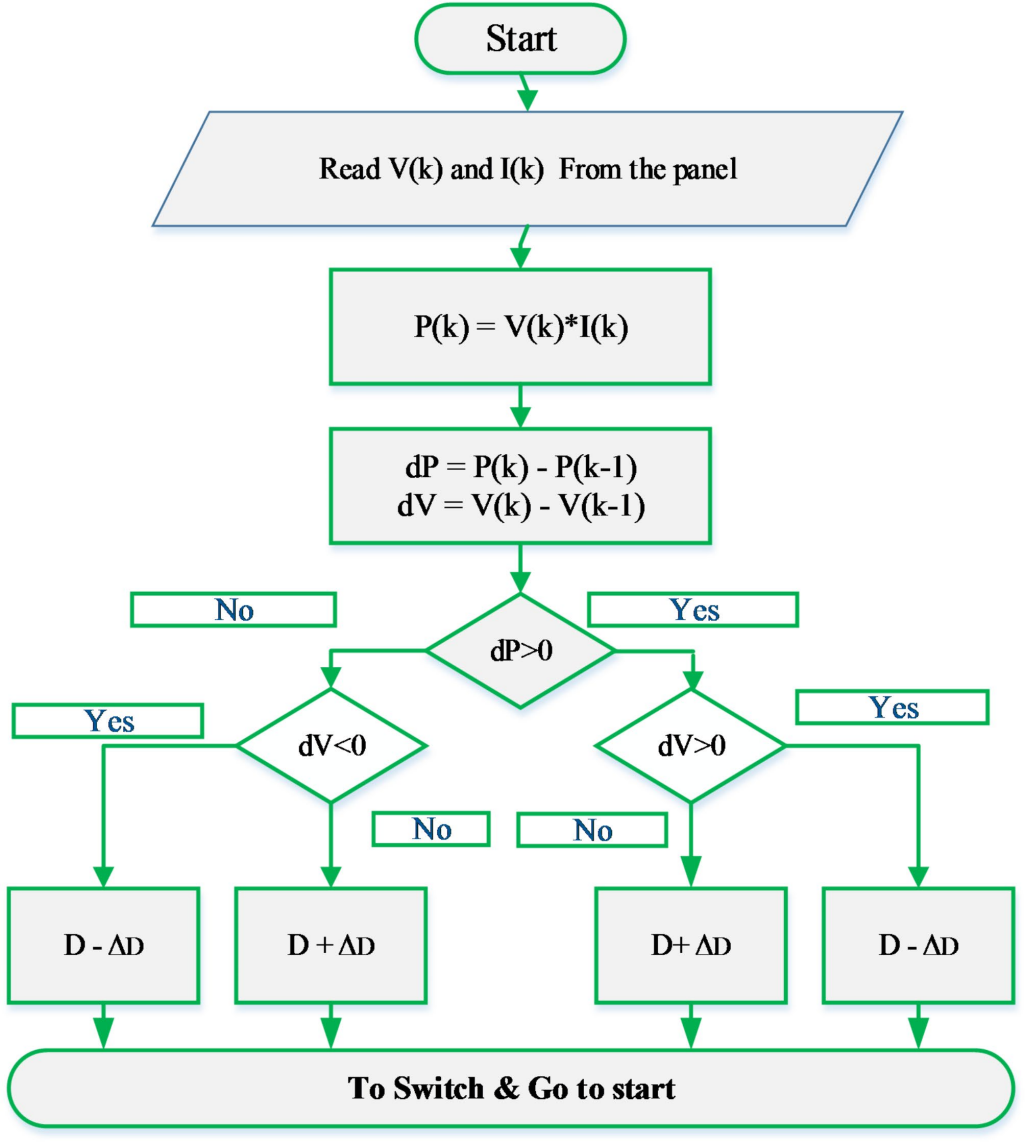
Flowchart of P&O MPPT technique.

The variables $\:{I}_{pv},\:{I}_{ph},\:{I}_{r},\:{I}_{rr},\:{V}_{pv},\:{V}_{oc}\:\:\:$represent various aspects of the PV cell, including its output current, light-generated current, reverse saturation current, diode reverse saturation current, PV array voltage, and open-circuit voltage, respectively. Additionally, $\:{R}_{s}$ denotes the series resistance, while $\:{R}_{p}$ signifies the shunt resistance. The Boltzmann constant is denoted by K, and T represents the cell temperature, with N representing the insolation (W/m2). Furthermore, $\:{I}_{sc}$ denotes the short circuit current at standard test conditions (STC), and Eg represents the bandgap energy, which is 1.12 eV for Si. The electrical characteristics of the PV array utilized in this investigation are presented in Table 2. Furthermore, Fig. 6 illustrates the flowchart of the Perturb and Observe (P&O) Maximum Power Point Tracking (MPPT) algorithm utilized to monitor the maximum power of the photovoltaic modules.

**Table 2:** Comparison of Inverter Topologies.

Inverter Topology	Voltage Gain	Component Stress	Efficiency	Applications	References
Two-Stage DC-DC-AC	High (separate boost and inverter stages)	Moderate (more components)	Moderate (conversion losses)	Suitable for large-scale PV systems	^19,20^
Single-Stage DC-AC (VSI)	Moderate	High	Moderate	Cost-effective for small systems	^6,7^
Single-Stage QB-SSI	High (integrated boost and inverter stages)	Low	High (reduced component count)	Optimized for EV charging and grid connection	^9,10^

**Fig. 7:**
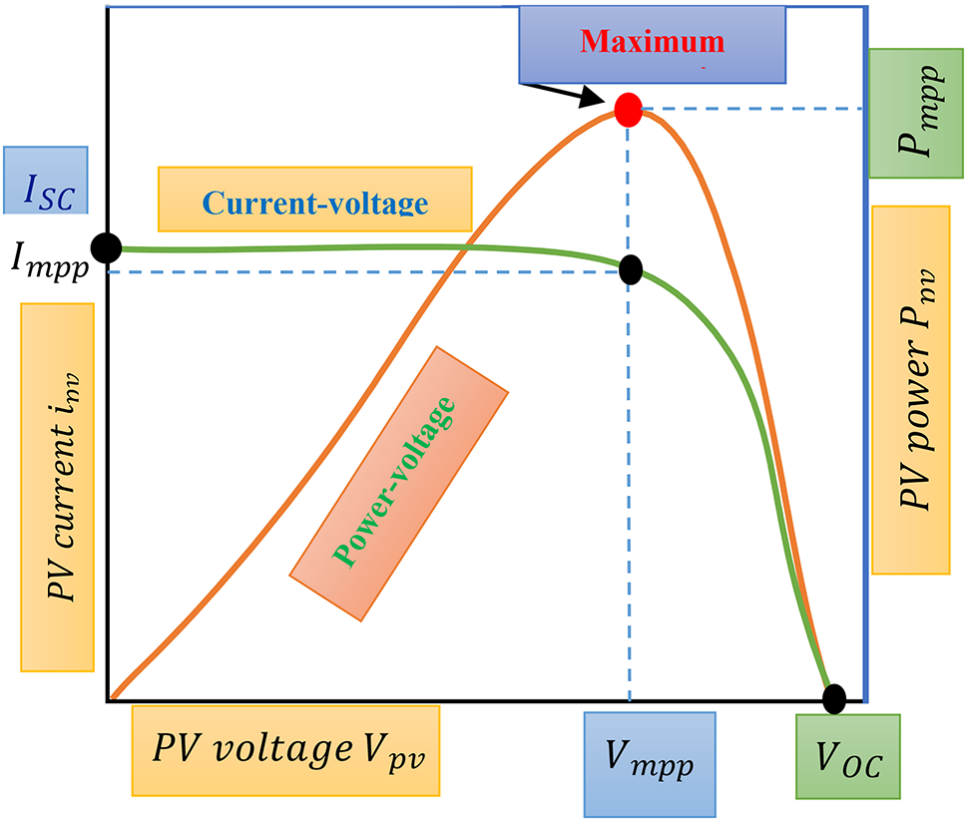
Power-voltage (P-V) characteristic of PV modules.

One of the commonly employed techniques is the perturb and observe method, utilized for maximizing the power output of PV modules. This approach is renowned for its efficiency and straightforward structure^18^. Figure 7 provides an illustration of a typical power-voltage (P-V) curve (in green) and current-voltage (I-V) curve (in blue) for the PV panel. It demonstrates that the maximum power point (MPP) occurs at the red point on the P-V curve, representing the point where the maximum energy, denoted as$\:{P}_{MPPT}$, can be extracted. Furthermore, the I-V curve indicates the corresponding voltage ($\:{V}_{MPPT}$) and current ($\:{I}_{MPPT}$) at the MPP. Notably, on the left side of the MPP, the PV operating voltage exhibits an almost linear relationship with the output energy, while beyond the MPP voltage, the power diminishes as the PV voltage increases. The power output of the PV modules is continuously evaluated and compared to previously recorded values, as the operating voltage of the PV modules undergoes incremental changes in one direction initially. To maintain the PV module’s operating point at the MPP, MPPT control involves continuous adjustment of the duty cycle provided to the boost DC-DC converter’s controller.

### Modulation

The proposed Quadratic-Boost Inverter (QBI) can be modulated using various PWM schemes commonly used for standard (VSIs). However, the modified unregulated SVPWM (MSVM) and regulated SVPWM (RMSVM) schemes, introduced in^2,16^ respectively, Both Sinusoidal Pulse Width Modulation (SPWM) and Space Vector Modulation (SVM) are acknowledged as the conventional modulation techniques for all extended-boost-based Split-Source Inverters (SSIs) owing to their significant advantages:

1. These strategies guarantee constant duty cycles for the charging and discharging of the inductor, leading to decreased input current ripples and voltage stresses. This characteristic enhances overall system performance and efficiency.
2. The MSVM and RMSVM schemes offer comparable performance to that of a two-stage topology. This means that the proposed QBI, when modulated using these schemes, can achieve similar functionality and performance as traditional two-stage inverters.

By adopting the MSVM or RMSVM schemes for modulating the QBI switches, the boosting and inversion functions of the inverter are effectively controlled. These modulation techniques provide benefits in terms of reduced current ripples, lower voltage stresses, and compatibility with established standards in extended-boost-based SSIs. The MSVM and RMSVM schemes are preferred choices for achieving optimal performance and efficiency in the operation of the QBI. Within the regulated modulation scheme, the (QB-SSI) can be perceived as two separate converters: a quadratic boost DC-DC converter and the (VSI). As a result, the conventional control modes commonly used in VSI systems can be implemented in the QSI as well.

**Fig. 8:**
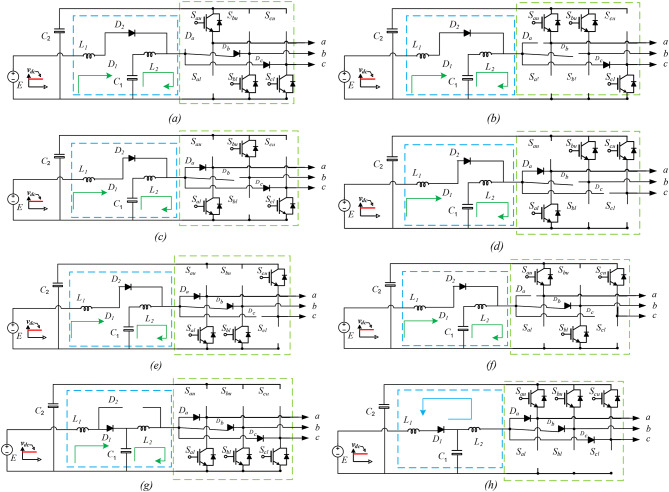
The modulation scheme employed in the Quadratic-Boost Split-Source Inverter.

Referring to Fig. 8, it can be observed that the upper and lower switches for each leg of the B6 circuit undergo commutation once during every half switching cycle, ensuring synchronized operation. This commutation process is characterized by variable duty cycles, akin to those utilized in conventional Voltage Source Inverters (VSIs), where the switching pattern dynamically adjusts to optimize performance. By leveraging this approach, the regulated modulation scheme effectively manages the switching sequences of the Quasi-Switched Inverter (QSI), ensuring stable voltage boosting, reduced switching losses, and improved power quality. Table 3 presents a summary of the comparison results among the analyzed topologies for q-ZSI, SSI, and QB-SSI, encompassing factors such as boosting factors, voltage stresses on capacitors and semiconductors, as well as the AC-voltage gain of various topologies.

**Table 3:** Illustrates a comparison between the proposed quadratic-Boost split-source inverter (QB-SSI) and the conventional full-bridge q-ZSI and SSI.

Inverter features	q-ZSI [6]	SSI [2]	QB- SSI
No, active switches	6	6	6
No, diodes	1	3	5
No, inductors	2	1	2
No, capacitors	2	1	2
THD	Low at low-voltage gains	Low at high-voltage gains	Low at high-voltage gains
Charging duty cycle, $\:{D}_{ch}$	1-M	M	M
Boosting factor B	$\:\frac{1}{\left(1-2{D}_{sh}\right)}$	$\:\frac{1}{\left(1-{D}_{ch}\right)}$	$\:\frac{1}{{\left(1-{D}_{ch}\right)}^{2}}$
PWM Complexity	Complex	Simple	Simple
Input current waveform	Continuous	Continuous	Continuous
DC- voltage	pulsed	Continuous	Continuous
Voltage gain	$\:\frac{M}{\left(\sqrt{3}\left(1-M\right)\right)}$	$\:\frac{M}{\left(\sqrt{3}\left(1-M\right)\right)}$	$\:\frac{M}{\left(\sqrt{3}{\left(1-M\right)}^{2}\right)}$

By treating the QB-SSI as separate converters and employing standard control techniques, the regulated modulation scheme offers familiarity and compatibility with established control strategies used in VSI systems. This simplifies the control implementation and facilitates the integration of the QSI into existing power conversion systems.

### RMSV modulation scheme

The Regulated RMSV Modulation for the QB-SSI modulation scheme, as depicted in Fig. 4^2^, is an advanced space vector modulation (SVM) strategy designed to optimize voltage gain, reduce switching losses, and enhance dynamic performance. Unlike conventional SVM, it incorporates a regulated reference voltage that dynamically adjusts the modulation index to improve the inverter’s efficiency and stability under varying operating conditions. By strategically selecting switching states, it minimizes total harmonic distortion (THD) and common-mode voltage (CMV), thereby reducing electromagnetic interference (EMI) and leakage currents—crucial for applications such as electric vehicles and renewable energy systems. The boosting capability is integrated within the QB-SSI topology, eliminating the need for an external DC-DC converter while ensuring high output voltage with improved power quality. Additionally, this modulation scheme enhances thermal management and system reliability by reducing high-frequency switching transitions, making it particularly effective in industrial motor drives, smart grids, and high-efficiency power conversion applications. This modulation scheme utilizes a single control parameter known as the modulation index (M), This facilitates interdependent control of both the AC and DC sides of the system. It is crucial to emphasize that the peak value of max($\:{v}_{a}^{*}$,$\:{v}_{b}^{*}$,$\:{v}_{c}^{*}$) remains constant at one. The Pulse Width Modulation(PWM) approach employed in this scheme is based on space vector modulation. Through the control of current in a rotating reference frame, this method produces a reference voltage. As discussed in the preceding section, a three-phase Split-Source Inverter (SSI) system offers a total of eight states, including six active states and two zero states. These eight states can be visualized as vectors that form a hexagon shape. The hexagon is divided into six sectors, each spanning 60 degrees. Within each sector, specific combinations of active and zero states are utilized to generate the desired output voltage. To generate a three-phase sinusoidal voltage reference vector, the technique of SVPWM is employed. This involves switching between the two adjacent active states and the zero state. By skilfully transitioning between these states, the SVPWM generates the desired voltage waveform with high accuracy and efficiency^23^.

The regulated RMSV modulation scheme provides precise control over the QB-SSI, allowing for efficient conversion of the input power. By adjusting the modulation index (M), the ac and dc sides of the system can be controlled simultaneously. This modulation scheme enables generation of high-quality three-phase sinusoidal voltages, meeting the desired performance requirements for grid-connected applications. Moreover, the common DC rail in this topology plays a crucial role in minimizing common-mode noise, which is essential for grid-connected applications. It is maintaining a consistent DC reference across the system to reduce voltage fluctuations that contribute to electromagnetic interference (EMI). This feature enhances the stability and reliability of the system under varying operational conditions^39^.

## Simulation results and analysis

The employed closed-loop arrangement utilizes the proposed regulated RMSVM (Regulated Reference Modulation for Quadratic-Boost Split-Source Inverters) method, incorporating both input power control and grid-side control as integral parts of the overall control strategy. This approach employs two independent control variables: The system involves two key parameters: The system involves two key parameters: the duty ratio $\:{D}_{O}$, regulating the input power on the DC side, and the modulation index M, overseeing the input power on the AC side.

To achieve MPPT, the input current controller is employed, regulating the duty ratio parameter $\:{D}_{O}$. The effectiveness of this controller is demonstrated through model simulations. In the simulations, a nominal voltage source with a rated voltage of 50 V is used to represent the dc input source, switching frequency ($\:{f}_{sw}$) 10 kHz, input power ($\:{p}_{out}$) 12 kW.

Additionally, the nominal grid voltage is adjusted to 110 $\:{V}_{rms}$ through the utilization of a grid simulator. The system ratings and the specifications of the PV array remain unchanged, are provided in Table 4.

**Table 4:** PV Module parametes.

Parameters	Values	Parameters	Values
Diode ideality factor	0.98117	Light-generated current IL (A)	7.865 A
Current at maximum power point Imp (A)	7.35 A	Series resistance Rs (ohms)	0.394
Cells per module (Ncell)	60	Open circuit voltage Voc (V)	36.3 V
Maximum Power (W)	213.15 W	Voltage at maximum power point Vmp (V)	29 V
Shunt resistance Rsh (ohms)	7.8649	Short-circuit current Isc (A)	7.84 A

By utilizing the regulated RMSVM approach and implementing a closed-loop configuration, the proposed control strategy ensures efficient power conversion and enables precise control over the input power on both the dc and ac sides of the system. This enables effective maximum power point tracking for the PV array and allows for optimal utilization of the available solar energy.

To regulate the voltage gain, Fig. 2 illustrates the utilization of a coupled inductor, enabling the attainment of a high voltage gain through the adjustment of the turn ratio. However, this configuration is susceptible to high-voltage stress on the main switch due to the presence of leakage inductance in the coupled inductor. To address this issue, the proposed converter integrates passive clamping circuits to mitigate the high-voltage stresses. Consequently, it becomes feasible to employ low-voltage-rating switching devices without incurring additional power losses from the snubber circuit. Specifically, a MOSFET with a low Rds (on) value can be employed for this purpose. Furthermore, the reverse-recovery problem associated with the output diode can be alleviated by leveraging the leakage inductance, thereby enhancing the overall power efficiency.

**Fig. 9:**
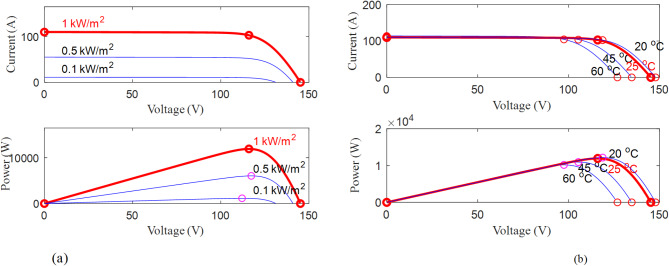
Illustrates the I-V characteristics of the module obtained using parameter estimation methods. The measurements were conducted by varying the temperature while keeping the irradiance constant at 1000 W/m2. Additionally, the I-V characteristics were also measured under different irradiance levels and at a temperature of 25 °C.

Figure 9 provides crucial insights into the performance of a photovoltaic (PV) module under different operating conditions by illustrating its I-V characteristics. Figure 9(a) depicts the I-V characteristics under varying irradiance levels while maintaining a constant temperature of 25 °C. As the irradiance increases, the short-circuit current ($\:{I}_{SC}$​) rises proportionally since higher sunlight intensity generates more electron-hole pairs in the semiconductor material. However, the open-circuit voltage ($\:{V}_{OC}$​) shows only a slight increase, as it depends logarithmically on the photocurrent. This indicates that irradiance primarily influences the current output rather than the voltage, directly affecting the power generation capacity of the module. On the other hand, Fig. 9(b) presents the I-V characteristics under different temperature conditions while keeping the irradiance constant at 1000 W/m². Unlike irradiance, temperature variations have a more pronounced effect on the voltage rather than the current. As temperature increases, the open-circuit voltage ($\:{V}_{OC}$​) decreases significantly due to an increase in carrier recombination and a reduction in the bandgap energy of the semiconductor material. In contrast, the short-circuit current ($\:{I}_{SC}$​) experiences only a slight increase with rising temperature, which is relatively negligible compared to the voltage drop. This results in a reduced maximum power output at higher temperatures, emphasizing the importance of thermal management in PV system applications. By analyzing these I-V characteristics, one can better understand the module’s behavior under different environmental conditions, which is crucial for optimizing its efficiency, implementing effective Maximum Power Point Tracking (MPPT) techniques, and ensuring the long-term reliability of the PV system. The ability to predict the module response to fluctuations in irradiance and temperature is essential for designing robust and efficient solar energy systems that maximize energy yield under varying climatic conditions.

The convergence between the measured and obtained I-V characteristics confirms the accuracy and reliability of the experimental results. Such analysis aids in understanding the behavior of the PV module under real-world conditions, enabling better system design and performance optimization.

**Fig. 10:**
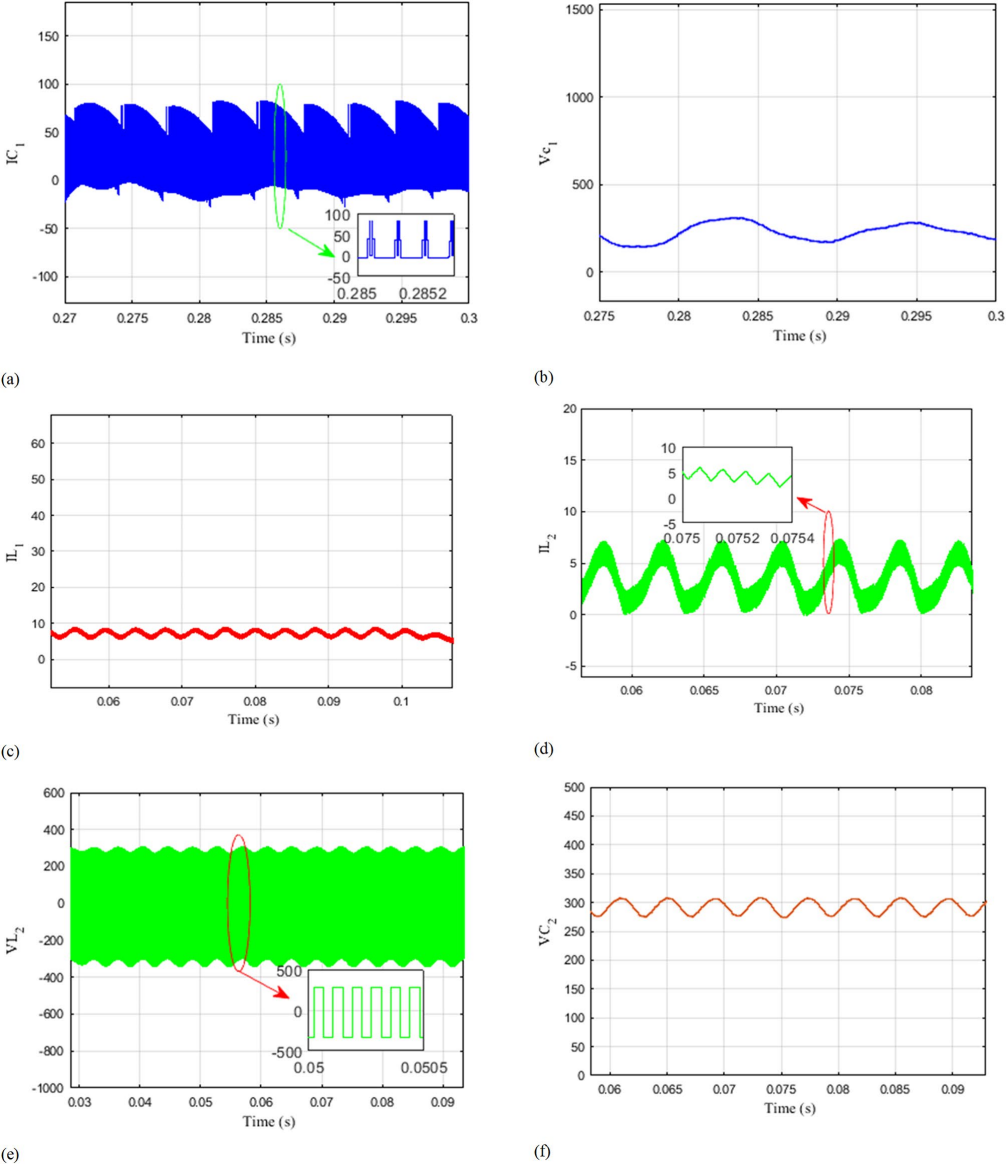

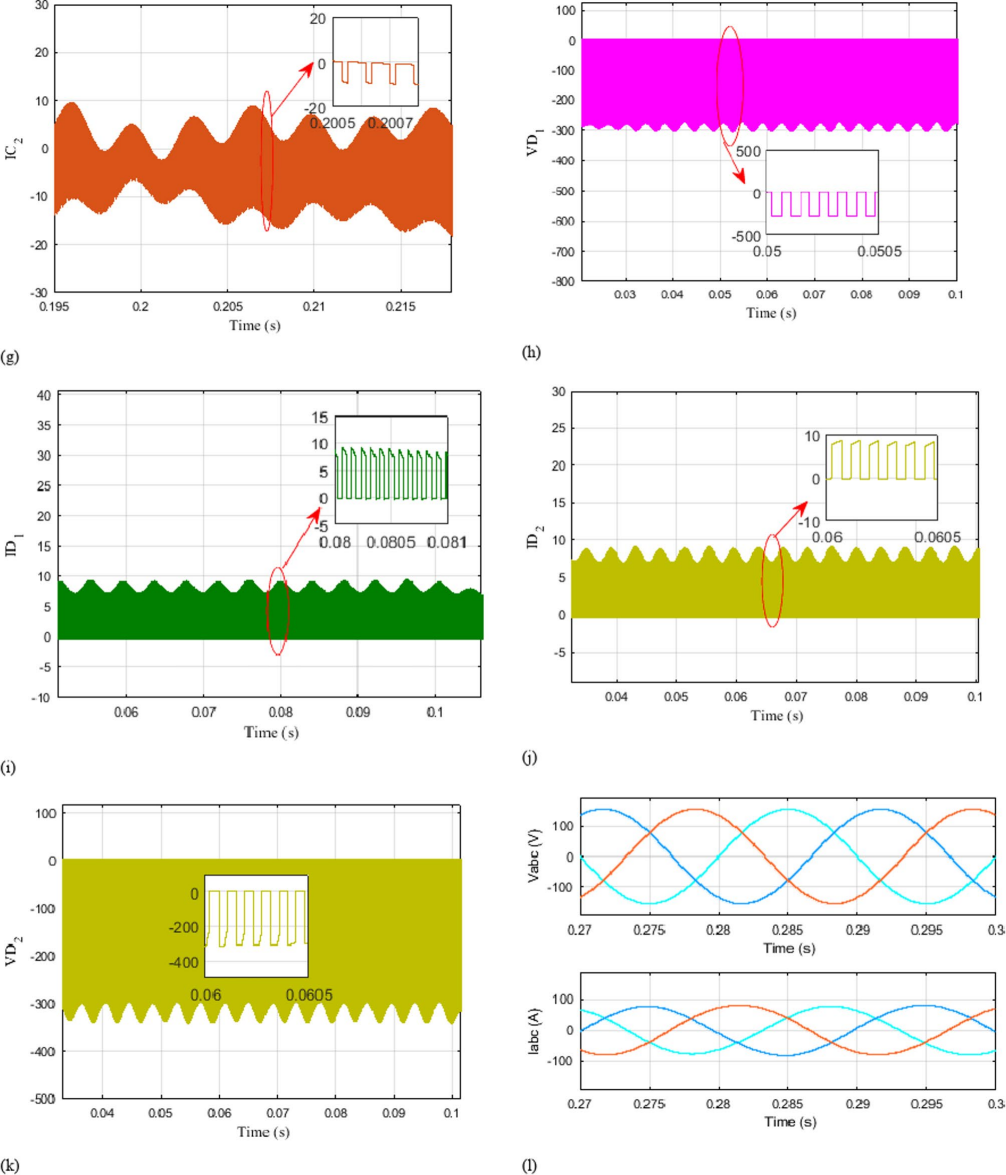
Simulation outcomes encompassing voltage and current outputs, along with detailed 1-millisecond snapshots of the waveforms across capacitors C1, C2, inductors L1, and L2, and diodes$\:{D}_{1},{D}_{2}$.

Figure 10.L illustrates the waveforms of grid voltage and current. It is noticeable that both the grid currents and the input DC current are proficiently regulated throughout the entire transient duration. The waveforms demonstrate the successful regulation and stability of the grid currents, ensuring that they remain within desired limits. Additionally, the input DC current is also accurately controlled, maintaining a stable and consistent behavior. For a more in-depth analysis of the three configurations, different numerical simulation via MATLAB/Simulink® are performed, the parameters used are listed in Table 2.

Figure 10 presents simulation results depicting the voltage and current waveforms of various components, including $\:{\text{D}}_{1},\:{\text{D}}_{2},\:{\text{C}}_{1},\:{\text{C}}_{2},\:{\text{L}}_{1},\:\text{a}\text{n}\text{d}\:{\text{L}}_{2}.$ In addition, close-up views of the waveforms with a time scale of 1 millisecond are included.

By examining the voltage and current waveforms of components ($\:{\text{D}}_{1},\:{\text{D}}_{2},\:{\text{C}}_{1},\:{\text{C}}_{2},\:{\text{L}}_{1},\:\text{a}\text{n}\text{d}\:{\text{L}}_{2}$ ), it’s possible to assess component stress levels and power losses. This information is instrumental in optimizing component selection and operating conditions to improve system efficiency and longevity. The extended analysis of Fig. 10 and the associated waveforms, coupled with numerical simulations and parameter transparency, provides a comprehensive understanding of the system’s performance, control strategies, and dynamic behaviors. These insights are crucial for optimizing system design, control algorithms, and component selection, ultimately leading to a more efficient and reliable power conversion system.

The close-up views of waveforms with a 1-millisecond time scale capture transient behaviors in detail. This includes rapid changes during switching events or load variations. Analyzing these transients is essential for evaluating the system’s dynamic response, ensuring it remains stable and robust under dynamic conditions. These simulation results offer a detailed insight into the behavior and performance of the mentioned components. By examining the voltage and current waveforms, it is possible to observe the switching patterns, transient responses, and overall characteristics of the components. The close-up views with a time scale of 1 millisecond provide a more detailed examination of the waveforms, allowing for a more accurate analysis of the rapid changes and dynamics occurring within the components. By studying these simulation results, it becomes possible to assess the performance, efficiency, and reliability of the system. It enables the identification of any potential issues, such as voltage spikes, current fluctuations, or any other abnormalities that may affect the overall operation of the components.

## Experimental results

**Fig. 11:**
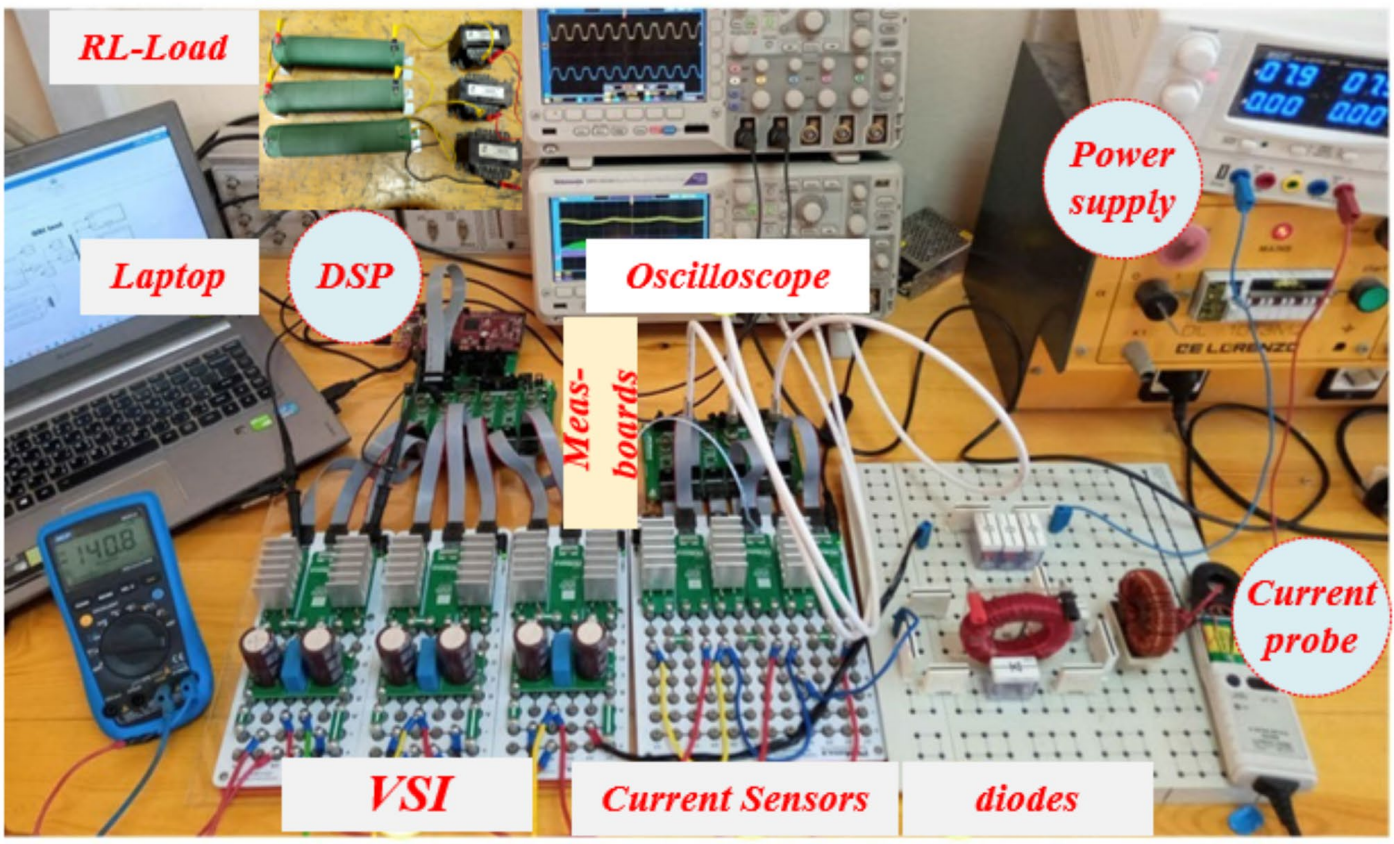
Experimental Setup Photograph.

This section elaborates on the experimental setup and the measurements conducted on a scaled-down prototype of the Proposed SSI inverter. A photograph of the experimental setup is presented in Fig. 11. The passive elements on the DC side were chosen to align with those utilized in the simulation study, ensuring consistency between theoretical and experimental analyses. The inverter output terminals were connected to a star-configured inductive load to replicate practical operating conditions. The principal parameters of the experimental system are outlined in Table 5.

**Table 5:** Experimental parameters.

Parameter	Value
Supply voltage	20 V
DC-side passive elements	$\:{L}_{1}\:=\:{L}_{2}\:=\:1.25mH$ $\:{C}_{1}\:=\:{C}_{2}\:=\:120\mu\:F$
Three-phase load	$\:11\:\varOmega\:\:\&\:5\:mH$
Output frequency	$\:50\:Hz$
Switching frequency	$\:20\:kHz$
Dead time	$\:2\:\mu\:s$
DSP	$\:TI-F28379D$

The cost-effective LAUNCHXL-F28379D development kit is utilized to implement the QBI modulation strategy, enabling the generation of a 50 V output line voltage at a frequency of $\:50\:Hz$. The experimental analysis of the proposed quasi-balanced inverter (QBI) highlights its robust performance and alignment with theoretical predictions.

**Fig. 12:**
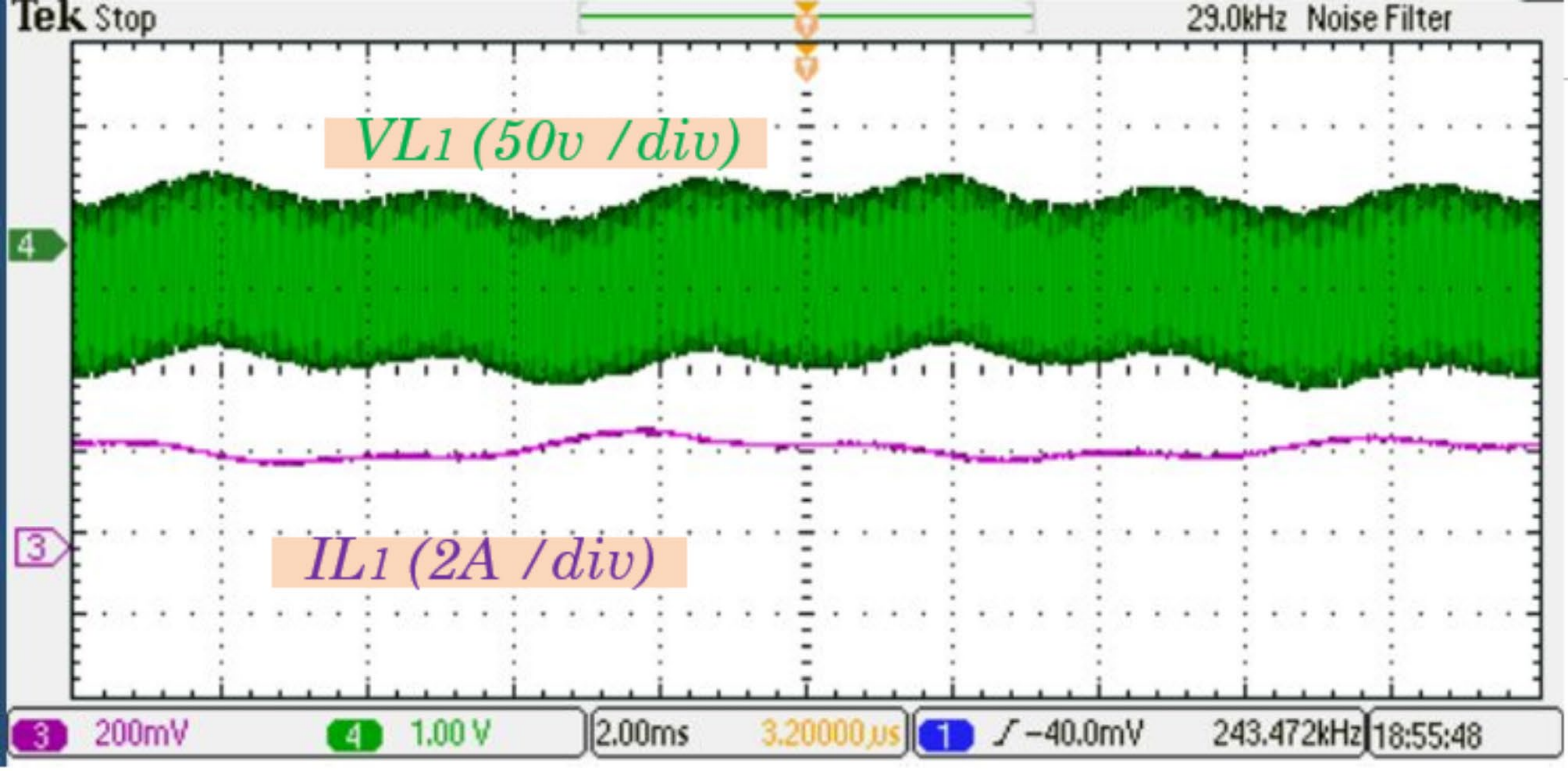
Experimental results for the inductor $\:{L}_{1}$ voltage and current waveforms.

**Fig. 13:**
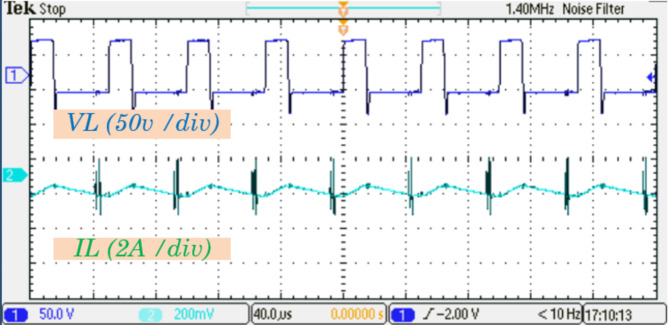
Experimental results zoomed-in views of the inductor $\:{L}_{1}$ voltage and current waveforms.

Figure 12 shows the voltage and current waveforms of inductor$\:\:{L}_{1}$, demonstrating the inductor dynamic response during operation. To provide a more detailed perspective, Fig. 13 presents a zoomed-in view of these waveforms, showcasing intricate transitions and variations in the inductor behavior.

**Fig. 14:**
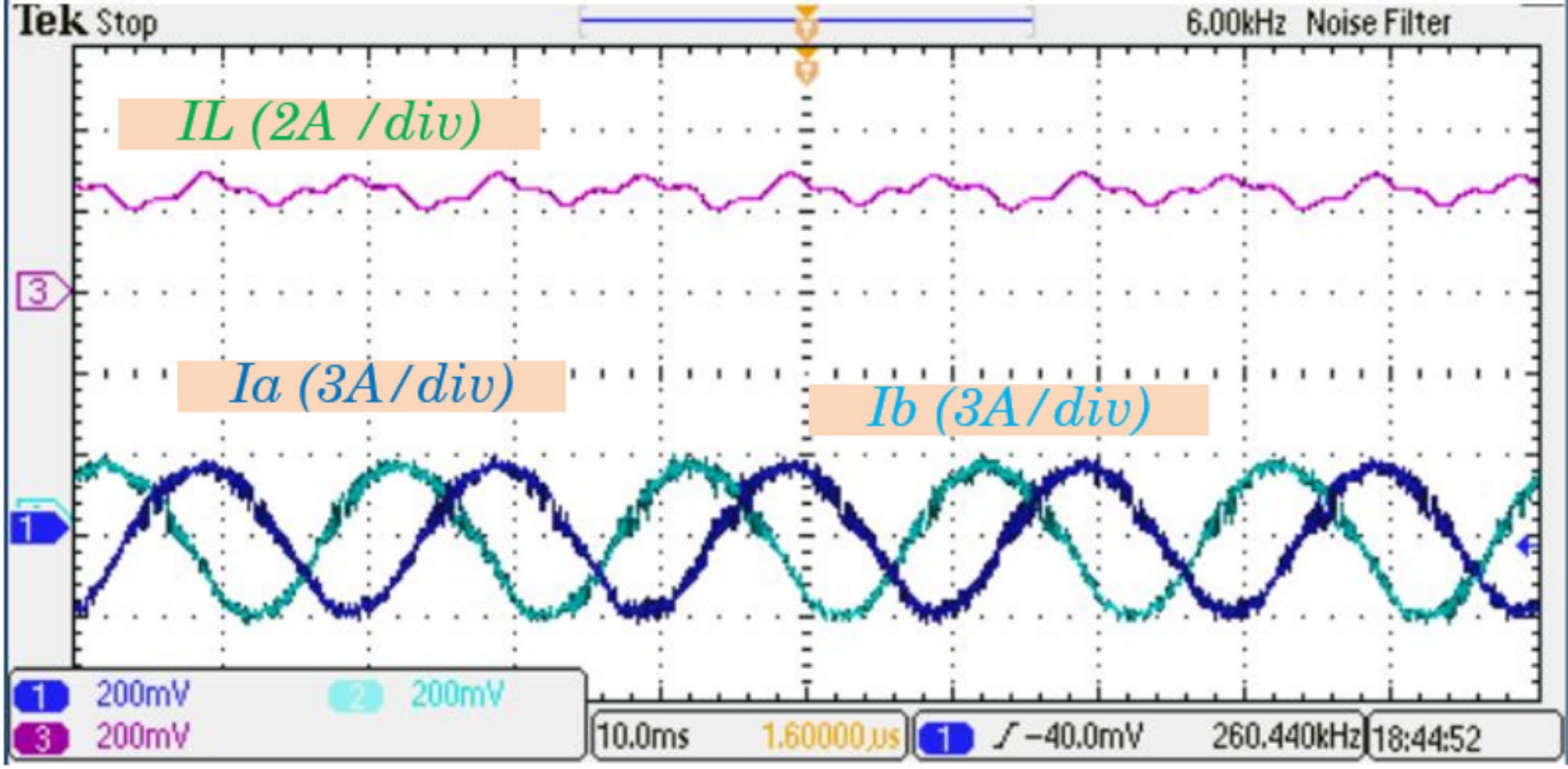
Experimental results for the inductor $\:{L}_{1}$ current and output currents $\:{I}_{a},\:{I}_{b}$ waveforms

**Fig. 15:**
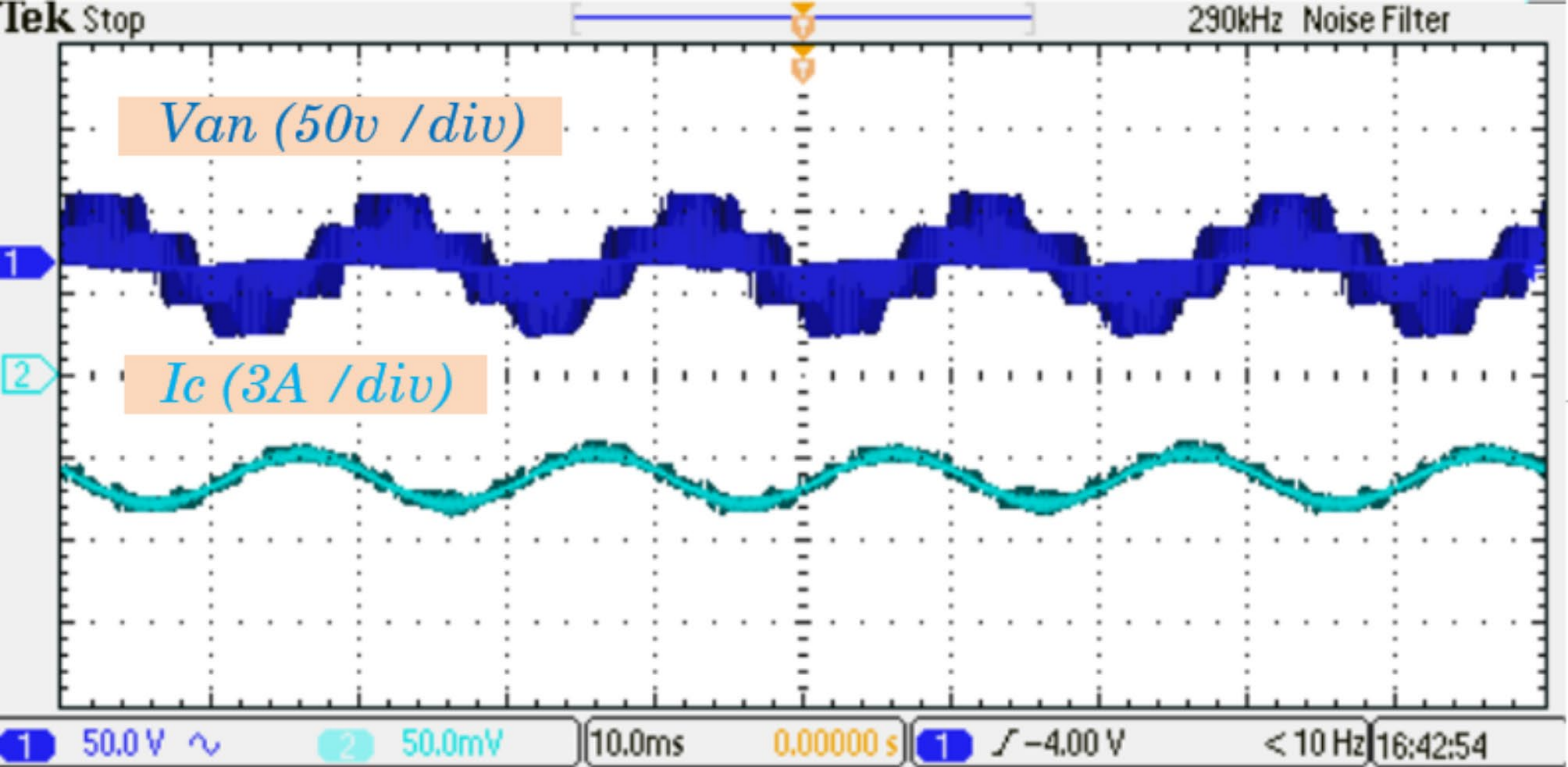
Experimental results for output phase voltage and output current $\:{I}_{C}$ waveforms.

Figure 14 further examines the current waveform of inductor $\:{L}_{1}$ alongside the output currents$\:{\:I}_{a},\:and\:{I}_{b}$. These output currents are nearly sinusoidal with minimal harmonic distortion, closely resembling the theoretical predictions. The exceptional waveform quality is largely attributed to the implementation of the Modified Space Vector Modulation (MSVM) schemes. MSVM enhances the inverter performance by optimizing switching patterns, thereby reducing harmonic distortions and ensuring smooth current profiles. In Fig. 15, the experimental results for the output phase voltage and the current waveform of $\:{I}_{C}$ are presented. These waveforms highlight the system ability to maintain consistent voltage and current profiles under dynamic operating conditions, further confirming its efficiency and stability.

The experimental results are in excellent agreement with the simulation outcomes and theoretical analysis. The near-sinusoidal output currents, low distortion levels, and stable voltage profiles demonstrate the practical feasibility and robustness of the proposed QBI system. These findings underscore its potential for efficient power electronic applications, validating its suitability for real-world implementation.

## Conclusions and perspectives

This paper introduces two enhanced SSI topologies that offer high voltage gains. These topologies, referred to as QB-SSI, leverage auxiliary inductors, capacitors, and diodes to amplify the boosting capability of the basic SSI. Consequently, there is a notable increase in both the dc-boosting factor and the ac voltage gain. The paper adopts a modified (MSVM) approach to minimize the dc-side ripples. Additionally, the paper provides a comprehensive guideline for designing a closed-loop controller. Furthermore, a decoupled control strategy incorporating MPPT is proposed, allowing independent control of the QB-SSI dc and ac sides. This feature proves advantageous for various applications. To improve switching characteristics and overall performance, the RMSVM scheme is employed for the proposed SSI-based topology, the feasibility of the proposed topology and the accuracy of the underlying theory are confirmed through simulation results derived from a MATLAB/Simulink model, as well as through experimental validation.

The introduction of these QB-SSI topologies opens exciting prospects for the field of power electronics and renewable energy systems. Their ability to significantly increase both DC-boosting and AC voltage gains can be particularly beneficial for applications requiring high voltage output. With the increasing prevalence of renewable energy sources such as solar and wind power, the demand for efficient energy conversion and storage solutions is on the rise. The QB-SSI configurations can significantly contribute to improving the performance and efficiency of these systems. The utilization of the MSVM approach to minimize DC-side ripples represents a notable advancement in power electronics. Reduced ripples contribute to better energy quality and can extend the lifespan of connected components. This technique has the potential to find applications beyond the scope of this paper, especially in systems where precise voltage control is critical. The proposal of a decoupled control strategy incorporating MPPT brings adaptability and versatility to SSI-based systems. This innovation can benefit various industries, including renewable energy generation, electric vehicles, and industrial automation, where efficient energy conversion and management are paramount.

## Data Availability

The datasets generated during and/or analyzed during the current study are available from the corresponding author on reasonable request.
